# Intralobular distribution of ovarian-like stroma in pancreatic mucinous cystic neoplasms: a discussion on its tumorigenesis

**DOI:** 10.1038/s41598-022-07416-9

**Published:** 2022-02-28

**Authors:** Yuki Fukumura, Yuko Kinowaki, Yoko Matsuda, Masaru Takase, Momoko Tonosaki, Masaaki Minagawa, Akio Saiura, Minoru Tanabe, Keiichi Okano, Yasuyuki Suzuki, Kota Kato, Takashi Yao

**Affiliations:** 1 Department of Human Pathology, School of Medicine, Juntendo University, Building A 10F, Hongo 2-1-1, Bunkyo-ku, Tokyo, 113-8421 Japan; 2 Division of Surgical Pathology, Tokyo Medical and Dental University Hospital, Tokyo, Japan; 3 Department of Pathology and Host-Defense, Faculty of Medicine, Kagawa University, Kagawa, Japan; 4 Department of Clinical Laboratory, Koshigaya Municipal Hospital, Saitama, Japan; 5 Department of Hepatobiliary-Pancreatic Surgery, School of Medicine, Juntendo University, Tokyo, Japan; 6 Department of Hepatobiliary and Pancreatic Surgery, Tokyo Medical and Dental University Hospital, Tokyo, Japan; 7 Department of Gastroenterological Surgery, Faculty of Medicine, Kagawa University Hospital, Kagawa University, Kagawa, Japan; 8 Department of Anatomy and Life Structure, School of Medicine, Juntendo University, Tokyo, Japan

**Keywords:** Oncogenesis, Medical research

## Abstract

Pancreatic mucinous cystic neoplasm (MCN) has two histological components: tumor epithelia and ovarian-like stroma (OLS). To examine the progression and changes in pancreatic MCNs, we analyzed the distribution, amount, immunohistochemical phenotype, presence of theca cells of OLS, and tumor epithelium in 45 surgically resected MCN cases, comparing them with tumor sizes. The OLS data of female MCN cases were also compared between those who were ≤ 51 years old and those > 51 years old to see the effect of menopause on MCN histology. Non-mucinous type epithelium was present in all low-grade MCNs, but its ratio decreased with tumor size (*p* < 0.001), suggesting that epithelial mucinous changes are a progression phenomenon. The intralobular distribution of OLS was observed in 28.8% of MCN cases and was related to smaller tumor size (*p* < 0.0001), suggesting intralobular involvement of early MCNs. The nuclear expression of β-catenin and the cytoplasmic expression of α-smooth muscle actin (SMA) was observed in almost all OLS. OLS tended to be lesser among female cases aged > 51 years than those ≤ 51 years old, however it did not reach statistical significance. This is the first study to show the intralobular distribution of OLS.

## Introduction

Mucinous cystic neoplasm (MCN) is a cyst-forming mucinous tumor that develops mainly in the ovary, pancreas, and liver but rarely in the mesentery, retroperitoneum, and spleen^1–3^. In the pancreas, MCN primarily occurs in the pancreatic body or tail of middle-aged women, does not communicate with the pancreatic duct, and, unlike branch duct intraductal papillary mucinous neoplasms (IPMNs), is always a single lesion^4,5^. MCN has two histological components: tumor epithelia and ovarian-like stroma (OLS). The OLS expresses ER and PgR, sometimes containing inhibin α-positive theca cells and is the most specific histological feature of MCN^6–8^. Despite these specific pathological features of MCN, its tumorigenesis is not well known. There are currently two hypotheses regarding the origin of MCN: (i) pancreatic remnant/attachment of primordial germ cells during their migration to the gonad (ectopic ovarian tissue)^3,9–12^, and (ii) endodermal-derived epithelium and primitive mesenchyme in the pancreas and proliferation under the stimuli of female sex steroids^3,12^. Between these two theories, recent molecular studies have favored the pancreatic remnant hypothesis^9–11^. In this study, to examine the progression, alteration of histology and immunophenotype, and tumorigenesis of pancreatic MCNs, we analyzed the distribution, amount, immunohistochemical phenotype, presence of theca cells in the OLS, and pathological features of the tumor epithelium of MCN tumors.

## Results

Clinicopathological data of MCN patients, cysts, tumor epithelium, and OLS are shown in Supplementary Table 1. Overall, 45 patients were included in this study (mean age, 45.8 ± 14.0 years; range 22–78 years; 44 patients (97.8%) were women). Among the female patients, 30 were ≤ 51 years old and 14 were > 51 years old. The mean tumor size was 62.0 mm (range 12–150 mm). All MCNs, except one, were located at the pancreatic body or tail, and 33 (73.3%) were multilocular and 12 (26.7%) were monolocular. In ten cases (22.2%), the MCN was localized in one half (or smaller) of the cut sections, which belonged to the inferior half (n = 6), including the anterior-inferior (n = 2) and the posterior-inferior (n = 2) sections and the superior half (n = 4), including the anterior–superior section (n = 2). Eleven of the 45 MCN cases (24.4%) contained high-grade or associated invasive carcinoma components. Non-mucinous type epithelia were seen in all 34 low-grade MCN cases (100%), and the mean ratio of non-mucinous type was 48.0% (range 5–90%) in low-grade MCN cases.

**Figure 1:**
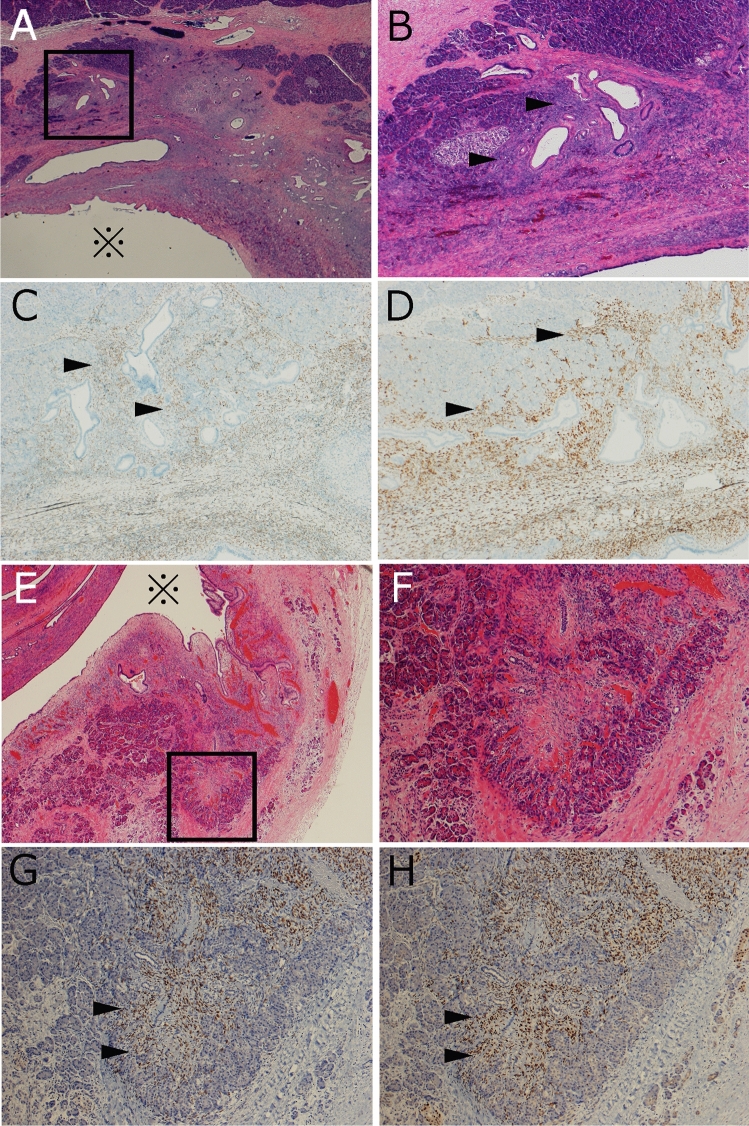
Intralobular distribution of ovalian-like stroma (OLS). (**A**–**D**) Mucinous cystic neoplasm (MCN) case 1. (**A**) MCN cyst (*) and intralobularly distributed OLS. (**B**) Higher magnification of the square region of 1A. (**C**) ER positivity in OLS. (**D**) PgR positivity in OLS. 1E-H. MCN case 4. (**E**) MCN cyst (*) and intralobularly distributed OLS. (**F**) Higher magnification of the square region of (**E**). (**G**) ER positivity in OLS. (**H**) PgR positivity in OLS. Arrow heads in (**B**–**D**) and (**F**–**H**) show intralobular OLS. (**C**, **G**) Immunohistochemistry for ER. (**D**, **H**) Immunohistochemistry for PgR.

All MCN cases contained OLS and were confirmed to be positive for both ER and PgR immunohistochemically, with 66.7% (30/45), 20.0% (9/45), and 13.3% (6/45) cases of MCN containing abundant, moderate, and scarce amounts of OLS, respectively. Distribution of OLS was as follows: cyst wall (C), 97.8% (44/45); septum of the cyst (S), 62.2% (28/45); inside the pancreatic lobule (IL), 28.8% (13/45); and perilobular area (PL), 6.7% (3/45). There was no OLS at extrapancreatic site (EX) observed for any of the MCN cases (Fig. 1). Theca cells were immunohistochemically confirmed with inhibin α expression and were present in 62.2% (28/45) of the MCN cases, and among the 28 MCN cases with theca cells, 92.9% (26/28) of them were located at C, 10.7% (3/28) were at S, 11.1% (5/45) were located at IL, and 7.1% (2/28) were at PL (Supplementary Table 1).

**Figure 2:**
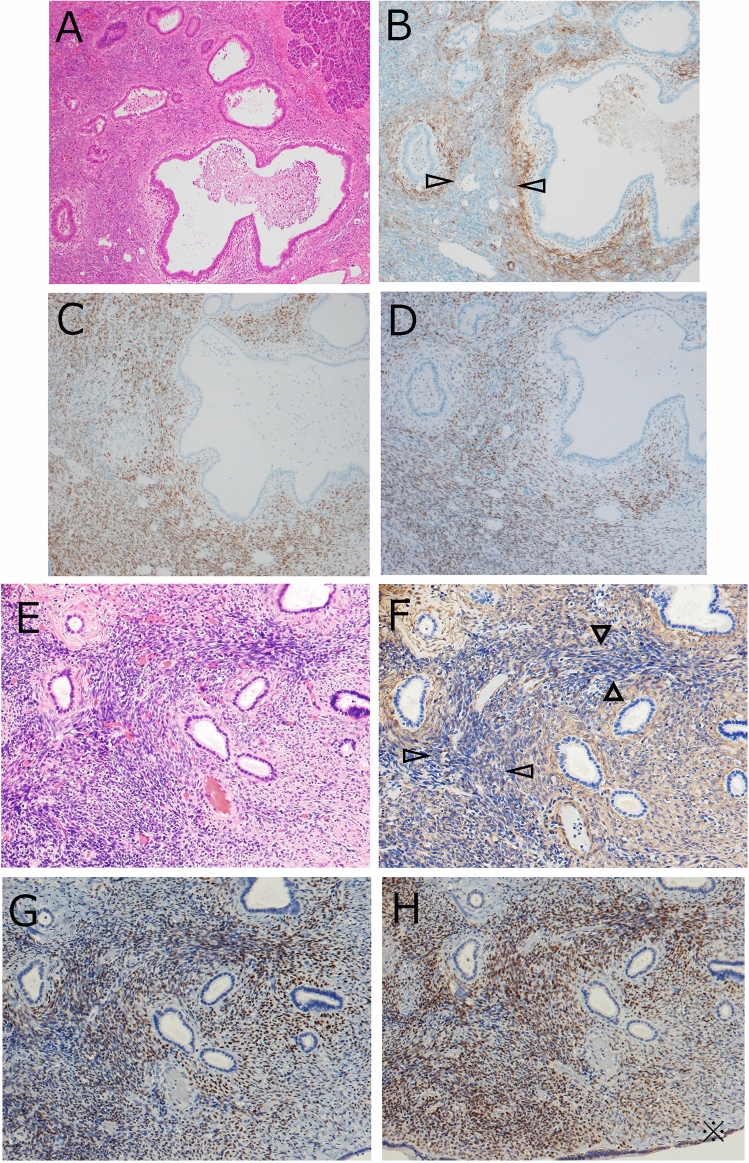
Occasional α smooth muscle actin (SMA)-negative status of ovarian-like stroma (OLS). (**A**–**D**) Mucinous cystic neoplasm (MCN) case 1. (**A**) OLS in the intralobular portion. (**B**) The OLS at the periductal part is α-SMA-positive and that with no association to dilated ducts is α-SMA -negative. C and D. The OLS is positive for ER (**C**) and PgR (**D**), irrespective of the distance from the ducts. 2E-H. MCN case 10. (**E**) OLS at the septum of the MCN cyst. (**F**) The OLS at the periductal part is α-SMA-positive and that with no association to dilated ducts is α-SMA -negative. (**G**, **H**) OLS is positive for ER (**G**) and PgR (**H**), irrespective of the distance from the ducts. Blank arrowheads in B and F show areas of α-SMA-negative OLS. (**B**, **F**) Immunohistochemistry for α-SMA, (**C**, **G**) Immunohistochemistry for ER, and (**D**, **H**) Immunohistochemistry for PgR.

**Figure 3:**
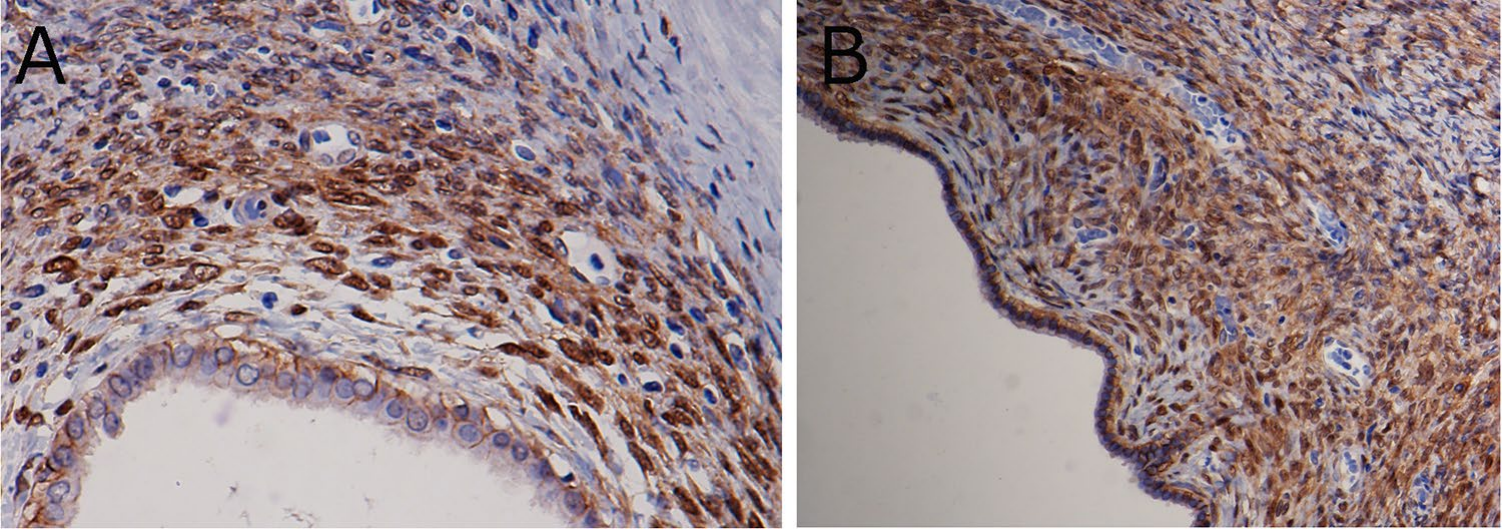
Nuclear accumulation of β-catenin in ovarian-like stroma (OLS). For both the intralobular OLS (**A**) and OLS at the cyst wall (**B**), nuclear accumulation of β-catenin was observed. [**A**, **B** Immunohistochemistry for β-catenin].

Immunohistochemically, most OLS were positive for α-smooth muscle actin (SMA) irrespective of OLS distribution sites or MCN size, whereas three MCN (6.7%) cases contained some areas composed of α-SMA-negative OLS. In these three cases, α-SMA-negative OLS was observed in the IL (n = 2), C (n = 1), and S (n = 1) (Supplementary Table 1). At IL, C and S, OLS surrounding the dilated pancreatic ducts or MCN cysts were α-SMA-positive, whereas OLS unassociated to the dilated pancreatic ducts were negative (Fig. 2). Nuclear β-catenin accumulation was observed in OLS and theca cells, but not in tumor epithelia at all locations (C, S, IL, or PL) of all MCN cases (Fig. 3).

Logistic analyses showed that higher histological grade (high or high grade with invasion) was related to larger tumor size (p = 0.004), and a higher ratio of non-mucinous type epithelium was related to smaller tumor size (*p* < 0.001) (Table 1). The IL-distribution of OLS was related to smaller tumor size (*p* < 0.0001) (Table 2). In addition, OLS tended to be more abundant in MCN of women ≤ 51 years old compared to those of women > 51 years old. however, this relationship was not of statistical significance (*p* = 0.194) (Table 3). There was no evident relationship between the existence of IL-OLS and patients’ age (women ≤ 51 years of age or in those > 51 years old) (*p* = 0.654).

**Table 1:** The relationship between clinicopathological data and tumor size of pancreatic MCN.

		*p *value (vs tumor size)*
Age		NS (*p* = 0.052)
Mean	45.8	
Range	22–78	
Cyst		NS (*p* = 0.304)
Monolocular	12	
Multilocular	33	
Grade		*p* = 0.004
Low	34	(R^2^ = 0.252, β = − 23.8)
High +	11	
Ratio of non-mucinous type		*p* < 0.001
Mean	48% (n = 34**)	(R^2^ = 0.37, β = − 77.3)
Range	10–90	

*p value, R^2^ score (contribution rate), and β-value (coefficient) by regression/logistic analysis were shown.

**Ratio of non-mucinous type epithelium was analyzed for low grade-MCN cases (n = 21).

MCN, mucinous cystic neoplasm.

**Table 2:** The relationship between OLS data and tumor size of pancreatic MCN.

		*p* value (vs tumor size)*
OLS amount		NS (*p* = 0.228)
Abundant	30	
Moderate	9	
Scarce	6	
OLS at IL		*p* < 0.0001
Present	14	(R^2^ = 0.29, β = 23.7)
Absent	31	
Theca cell		NS (*p* = 0.306)
Present	24	
Absent	21	

*p value, R^2^ score (contribution rate), and β-value (coefficient) by regression/logistic analysis were shown.

IL, inside the pancreatic lobule; MCN, mucinous cystic neoplasm; OLS, ovarian-like stroma.

**Table 3:** The relationship between OLS data and patients’ age in female pancreatic MCN.

	Age ≤ 51 (n = 30)	Age > 51 (n = 14)	*p* value
OLS amount			NS (*p* = 0.194)
Abundant	22	7	
Moderate	5	4	
Scarce	3	3	
OLS at IL			NS (*p* = 0.654)
Present	10	4	
Absent	20	11	
Theca cell			
Present	16	5	NS (*p* = 0.205)
Absent	14	9	

IL, inside the pancreatic lobule; MCN, mucinous cystic neoplasm; OLS, ovarian-like stroma.

## Discussion

The exact mechanism of tumorigenesis in MCN has yet to be elucidated. To examine its histological progression and possibly look into its tumorigenesis, we analyzed size-dependent histological and immunohistochemical changes of MCN in this study. We hypothesized that the smaller MCN group would have histological features characteristic of that of the early phase of MCN development. The relationship between the histological features and tumor size of the MCN was analyzed. To determine the possible relationship between menopausal status and the pathological features of OLS, data of MCN of female patients at ages ≤ 51 years and > 51 years were compared.

Our study showed that high-grade or high-grade with invasive carcinoma components are more often found in larger MCNs. The non-mucinous-type epithelium was seen in all low-grade MCN cases, and the ratio of non-mucinous epithelium among the entire epithelium was significantly higher in smaller MCNs. Our results showed that the non-mucinous epithelium was a common finding in pancreatic MCN and that smaller MCN contained more non-mucinous type epithelium, which is concordant with a previous study^13^. This suggests that the mucinous change of the epithelium is a “progression” phenomenon in pancreatic MCNs.

As for the location of OLS, we found that smaller MCNs more often had IL-OLS. The presence of OLS is the most specific histological feature of MCN, and OLS is located immediately beneath or associating with the neoplastic epithelium^14^. To the best of our knowledge, the IL-distribution of the OLS in pancreatic MCN has never been reported. In this study, 28.8% of our MCN cohort, or 75.0% of MCN cases sized ≤ 30 mm, contained IL-OLS. These IL-OLS are observed at clearly distant sites or have no association with the neoplastic epithelium. We propose two explanations for this: (1) IL-OLS is an early event or (2) a late event in MCN development. In (2), when OLS grows, the surrounding pancreatic lobules eventually become involved. However, we think the hypothesis that IL-OLS being a late event in MCN development is unlikely since IL-OLS was seen mostly in small MCN cases and at sites clearly distant from the neoplastic epithelium. Hence, the frequent intralobular distribution of OLS in small-size MCN suggests that early MCN lesions often involve pancreatic lobules and that some MCNs might be generated from pancreatic lobules, although it is our postulation merely from histological observational studies.

Since pancreatic MCN is usually detected or surgically resected when it is large, it is generally difficult to see where or in which part of the pancreatic parenchyma is MCN generated. Although it is well known that pancreatic MCNs do not communicate with pancreatic ducts, it is possible that MCNs may have communicated with small ducts, such as intralobular or intercalated ducts or acinus in the early phase of MCN development. An experimental study by Sano et al., reported the intralobular generation of pancreatic MCN^15^, wherein MCN with ER-positive and PgR-positive OLS were developed by introducing Wnt1 into pancreatic acinar cells and acinar cell progenitors in *LSL-Kras*^G12D^, *Ptf1a-cre* mice. The authors reported the importance of the interaction between acinar cells and OLS in MCN development.

In this study, ten MCN cases were small enough to localize in either half of the pancreatic cut section, and the locations of these ten cases were relatively diverse; six cases (60.0%) were in the inferior half portion of the pancreatic cut section and four cases (40.0%) in the superior, where two case was localized in the anterior/inferior, two in the posterior/inferior, and two in the anterior/superior quarter. No OLS was detected at the EX in this study, suggesting that early MCN may start at the site of the pancreatic parenchyma, mostly at the pancreatic body or tail, but not at the EX.

There are two hypotheses proposed for MCN development: (i) the pancreatic attachment of primordial germ cells during their migration to the gonad (ectopic ovary)^9–11^, and (ii) endodermal-derived epithelium and primitive mesenchyme proliferation in the pancreas with female sex steroids as stimuli^3,12^. We examined whether there were ectopic ovary or possible OLS precursors in normal pancreatic specimens, utilizing HE-stained and ER-stained specimens from 52 distal pancreatectomy cases (21 cases of neuroendocrine tumor, 21 cases of small pancreatic ductal carcinoma, five cases of serous cystic neoplasm, 5 cases of metastatic pancreatic carcinoma), and found that peri-acinar spindle or stellate-shaped cells as well as spindle fibroblastic cells around small and large ducts were positive for ER [Supplementary Figure 1]. However, no typical OLS cluster, showing densely packed spindle cells with sparce cytoplasm was observed in any case. With the fact that our OLS search in 52 cases of normal pancreatic body to tail specimens did not detect typical OLS, it can be proposed that OLS growth is a very rare event, and if it occurs, it may lead to the quick formation of a pancreatic cyst. More observational studies are necessary to confirm this. Although normal stromal cells are positive for ER, we considered the ER/PgR-positive stromal cells as OLS only when they showed distinctive histological features.

Regarding the amount of OLS, our study showed that MCN of women ≤ 51 years of age tended to harbor a higher quantity of OLS compared to those of women > 51 years of age, although this relationship did not reach statistical significance. This suggests that OLS might decrease in amount after menopause in female MCN cases. Since we wanted to determine the effect of menopause on the pathological features of OLS, we used the age of 51 as the cut-off point in this study, according to the reported average age for menopause^18^, since the menopausal status of most of the patients had not been recorded.

Using immunohistochemistry, nuclear expression of β-catenin for OLS was observed for all MCN cases and all OLS sites. Several studies have suggested the activation of Wnt signaling pathway in OLS and its contribution to MCN development^15–17^. Sano et al. have shown the activation of Wnt signaling pathway in OLS by introducing *Wnt1* and *KRAS*^G12D^ to the epithelial component in mice^15^. Fukushima et al. have shown the overexpression of *Wnt2B* and *secreted frizzled-related protein*, a moderator of Wnt pathway in OLS^16^ and Treek et al. have shown the upregulation of Wnt and the Hedgehog pathway in OLS^17^. This is in line with our result that all OLS showed nuclear accumulation of β-catenin irrespective of cyst size or OLS site and suggests consistent activation of Wnt pathway for OLS. The contribution of Wnt pathway activation in OLS may not only be for the early developmental phase but also the late phase.

The present study detected three (6.7%) MCN cases containing areas composed of α-SMA-negative OLS, although most OLS were α-SMA-positive. The immunoreactivity of OLS for α-SMA has been reported in several studies^1,7,14^. By analyzing OLS with electron microscopy and comparing the stroma of ovarian MCN and normal ovary, Shiono et al., have suggested that OLS harbors myofibroblastic features^1^. Several authors also showed OLS in MCN of the ovary as well as normal ovary stroma were positive for α-SMA^18^, suggesting α-SMA expression of OLS can be downregulated. Our observation that OLS with no association of dilated ducts was α-SMA-negative suggests that α-SMA-expression of OLS may be related to stimuli from ductal epithelium. Further studies with more MCN cases containing abundant OLS are needed to confirm our hypothesis.

**Figure 4:**
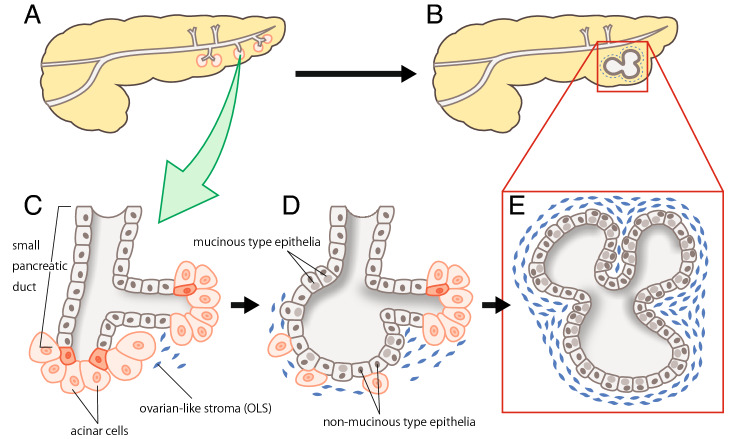
Schematic hypothesis on the development of mucinous cystic neoplasm (MCN). (**A**, **B**) Whole view of the pancreas containing early-phase MCN (**A**) and late-phase MCN (**B**). (**C**–**E**) Closer view of very early-phase MCN (**C**), early-phase MCN (**D**), and late-phase MCN (**E**).

In conclusion, our study showed an increase in histological grade and a decrease in the ratio of non-mucinous type epithelium with an increase in tumor size. We clarified that OLS can be observed at intralobular sites mainly in small-sized pancreatic MCNs, suggesting that some MCN may develop in the pancreatic lobules. One possible mechanism for the tumorigenesis of pancreatic MCN is shown in Fig. 4. The limitation of this study is related to its nature, which is an observational study of histology and immunohistochemical data. Thus, analyses of the size-dependent alterations in the genetic profile of OLS/tumor epithelia are required for future studies on pancreatic MCNs.

## Materials an methods

### Materials

Forty-five pancreatic MCN cases (44 cases in women and one case in a man) were analyzed. All patients underwent surgical resection, and 21, 21, and three patients underwent surgery at the Department of Hepatobiliary Pancreatic Surgery of Juntendo University Hospital, Tokyo Medical and Dental University Hospital, and Department of Gastroenterological Surgery of Kagawa University Hospital, respectively, between January, 2005 and December, 2021. MCNs were located at the pancreatic body or tail in 44 cases and at the pancreatic head in one case. OLS, confirmed by immunohistochemistry for ER and PgR, was observed in all cases. In each case, the entire pathological specimen was cut to 5-µm thickness and microscopic examination was performed with hematoxylin and eosin(HE)staining. Representative 1–3 sections per case were used for immunohistochemical evaluation.

### Methods

Clinicopathological data, including patients’ age, sex, and tumor site were collected. Tumor size, segment, and multi- or mono-locularity of the cyst were determined by macroscopic inspection of the tumor, where the maximal diameter of the MCN cyst was recorded for tumor size. When the tumor was small enough and localized to one-half of the cut section, we classified it based on which half the tumor belonged to (anterior, posterior, superior, or inferior). The histological grade of the MCN epithelium was classified as low, high, or high with associated invasive carcinoma, according to the World Health Organization classification^6^. The epithelium of MCN was categorized into mucinous or non-mucinous types based on a previous study^9^. The pancreatic intraepithelial neoplasia-like columnar cells with pale pink mucin were classified as mucinous type; whereas, flat to cuboidal to short columnar without obvious mucin or goblet cells were classified as non-mucinous type. The ratio of non-mucinous epithelia among the total cyst-lining epithelia was recorded in 10% increments by analyzing the entire tumorous lesion for each case, where the part in which no lining epithelia were seen was not counted. Since high-grade epithelial components of MCNs showed almost exclusively mucinous-type epithelium, the ratio of non-mucinous type epithelium was analyzed only for low-grade MCN cases.

### Assessment of OLS

The distribution and amount of OLS were determined by HE staining and immunohistochemistry for ER and PgR. The OLS was defined as the stroma cells similar to the ovarian stroma and showing positive for ER/PgR, immunohistochemically. The distribution of OLS was categorized into the C, S, IL, PL, and EX. When OLS was distributed in the acinar cell-containing areas, clearly separated from the MCN cyst, it was determined as IL, and when OLS was seen between pancreatic lobules or between the pancreatic duct and pancreatic lobule, it was determined as PL. The distinction between pancreatic duct and a part of the MCN cyst was as follows: when the epithelia-lined structure was far enough from the main MCN cyst, we considered it a duct, and when the structure was continuous or close enough to the main MCN cyst, we considered it a part of the cyst. The amount of OLS was classified based on the maximum thickness as either scarce (< 100 µm), moderate (≥ 100 µm but < 1000 µm), or abundant (≥ 1000 µm). The presence of theca cells was determined by HE staining and immunohistochemistry for inhibin α, and when present, their distribution was classified as C, S, IL, PL, and EX, similarly to the distribution of OLS. Immunohistochemistry for α-SMA and β-catenin (nuclear expression) was performed to determine the difference in the immunophenotype of OLS at different sites. This study was approved by the Ethics Committee of Juntendo University, Tokyo, Japan, in November 2018 (#J-2018076) and was performed in accordance with the Declaration of Helsinki. Informed consent was obtained from all participants.

### Statistical analysis

Single regression and single logistic analyses were performed for continuous data and ordinal/nominal data, respectively, to determine the relationship between tumor size and each clinicopathological feature of MCN.

The OLS data (OLS amount, presence at IL, and presence of theca cells) of MCN were compared using the chi-square test between younger (≤ 51 years old) and older patients (> 51 years old) to examine the effect of menopausal status on the OLS. Since exact data of patients’ menopausal status was not obtained for most patients, we divided the patients according to the average menopausal age^19^. JMP 14.2.0 statistical software (SAS Institute, Incorporation, Cary, NC) was used for statistical analyses.

## Supplementary Information

Supplementary Information 1.

Supplementary Information 2.
