# Supplementary material for: Intralobular distribution of ovarian-like stroma in pancreatic mucinous cystic neoplasms: a discussion on its tumorigenesis
Source: Sci Rep. 2022 Feb 28;12:3326. doi: 10.1038/s41598-022-07416-9 (PMC8885835; doi:10.1038/s41598-022-07416-9)
Supplement: Supplementary file 1 — Supplementary Information 1. [file 41598_2022_7416_MOESM1_ESM.pdf]

Intralobular distribution of ovarian-like stroma in pancreatic mucinous cystic neoplasms: A discussion on its tumorigenesis

Yuki Fukumura[1*], MD, PhD, Yuko Kinowaki[2], MD, PhD, Yoko Matsuda[3], MD, PhD, Masaru Takase[4,1], MD, PhD,

Momoko Tonosaki[1], MD, Masaaki Minagawa[5], MD, PhD, Akio Saiura[5], MD, PhD, Minoru Tanabe[6], MD, PhD,

Keiichi Okano[7], MD, PhD, Yasuyuki Suzuki[7], MD, PhD, Kota Kato[8], PhD, Takashi Yao[1], MD, PhD.

Supplementary Table 1. Clinicopathologic Findings of pancreatic MCN

Case	Age /Sex	Size[a]	MCN			Epithelium		OLS			
			Site[b]	Cyst[c]	Seg[d]	Grade[e]	nonM[f]	amount[g]	distribution[h]	theca[i]	SMA(-) area[j]
1	32/F	12	B	Mu	A, I	L	80%	+++	C, S, IL, PL	+. IL	IL
2	34/F	12	B	Mo	P, I	L	90%	+++	C, IL, PL	+, C, PL	

3	68/F	15	B	Mu	S	L	50%	++	C, S	+, C	
4	47/F	15	T	Mo	S	L	50%	+++	C, S, IL	+, C	C, IL
5	35/F	16	B	Mo	A, I	L	70%	+++	C, IL	+, C, IL	
6	58/F	18	BT	Mu	I	L	70%	+++	C, S, IL	+, C, IL	
7	39/F	20	B	Mu	P, I	L	80%	+++	C, S, IL	-	
8	42/F	20	B	Mo	A,S	L	80%	+++	C,S	+, C	
9	42/F	20	B	Mo	NA	L	70%	+++	C	+, C	
10	50/F	25	T	Mu	I	L	50%	+++	C, S	-	
11	51/F	30	T	Mu	NA	L	70%	+++	C, S, IL	-	
12	71/F	30	T	Mo	NA	L	90%	+++	C, IL	+, C	
13	63/F	30	T	Mu	NA	L	40%	+++	C, S, IL	+, C, S, IL	

14	58/M	32	BT	Mu	NA	H+I	NA	+++	C, S	+, C	
15	63/F	35	BT	Mu	NA	L	70%	+	C, S	-	
16	57/F	36	B	Mu	NA	L	80%	+++	C, IL	+, C, IL	
17	41/F	40	BT	Mu	NA	L	80%	+	C, S	-	
18	78/F	45	B	Mu	NA	L	20%	+	C, S	-	
19	53/F	45	BT	Mu	NA	H+I	NA	+	C, S	-	
20	30/F	45	BT	Mu	NA	L	20%	+++	C, S, IL	-	S
21	64/F	45	BT	Mu	NA	L	20%	+++	C, IL	+, C	
22	35/F	50	T	Mu	NA	H	NA	+++	C, S	+, C	
23	31/F	50	B	Mu	NA	L	70%	++	C, S, IL	-	
24	29/F	53	BT	Mu	NA	L	20%	+++	C	+, C	

25	51/F	55	H	Mu	NA	L	40%	+++	C, S	+, C
26	54/F	55	B	Mu	NA	L	20%	++	C, S	-
27	41/F	60	BT	Mu	NA	L	50%	++	C, S	-
28	46/F	60	BT	Mo	NA	L	30%	+++	C	+, C
29	27/F	62	T	Mu	NA	L	60%	+++	C	+, C
30	45/F	63	T	Mo	NA	L	30%	+	C, S	-
31	62/F	65	T	Mo	NA	H	NA	+++	C, S	-
32	63/F	80	B	Mo	NA	L	40%	++	C, S	-
33	41/F	85	T	Mu	NA	L	10%	+++	C	+, C
34	42/F	100	BT	Mu	NA	H+I	NA	++	S	+, S
35	36/F	105	BT	Mu	NA	L	20%	+++	C	+, C

36	29/F	110	BT	Mu	NA	L	30%	+++	C, S	-
37	42/F	110	BT	Mo	NA	L	20%	+++	C	+, C
38	59/F	110	T	Mu	NA	H	NA	+++	C	+, C
39	28/F	115	BT	Mu	NA	L	40%	+++	C, S	+, C
40	43/F	126	T	Mo	NA	H+I	NA	+	C, PL	+, C, PL
41	22/F	130	B	Mu	NA	H	NA	+++	C, S	+, C, S
42	22/F	130	B	Mu	NA	H	NA	+++	C	+, C
43	30/F	140	BT	Mu	NA	L	10%	++	C	+, C
44	45/F	140	T	Mu	NA	H+I	NA	++	C, S	-
45	60/F	150	T	Mu	NA	H+I	NA	++	C	-

[a]Size; maximum tumor diameter (mm), [b]Site; Tumor site, B, pancreatic body; T, pancreatic tail, BT, pancreatic body to tail, H, pancreatic head
[c]Cyst; Multi- or mono-locularity of the MCN cyst, Mu, multilocular; Mo, monolocular

[d]Seg; Pancreatic segment where MCN localized; A, anterior half, P, posterior half, S, superior half, I, inferior half

[e]Grade; Histological grade; L, low grade, H, high grade, H+I, high grade with invasion

[f]nonM; the ratio of non-mucinous type epithelium among the entire tumor epithelium (%) in low-grade MCN cases.

[g]Amount; The amount of OLS; +++, abundant; ++, moderate; +, scarce amount of OLS was seen

[h]distribution; The distribution site of OLS; C, cyst wall; S, septum, IL, intralobular, PL, periloblar.

[i]theca; the presence and distribution site of theca cells; C, cyst wall; S, septum; IL, intralobular; PL, perilobular

[j]SMA(-) area; the sites where SMA(-) OLS were seen, if present; IL, intralobular; C, cyst wall, S, septum
