# Supplementary material for: Intralobular distribution of ovarian-like stroma in pancreatic mucinous cystic neoplasms: a discussion on its tumorigenesis
Source: Sci Rep. 2022 Feb 28;12:3326. doi: 10.1038/s41598-022-07416-9 (PMC8885835; doi:10.1038/s41598-022-07416-9)

Supplementary Figure 1.
ER expression in normal pancreas.

ER is positive for stellate-shaped periacinar cells (Arrowheads) and for spindle-shaped periductal cells (Arrows).

A

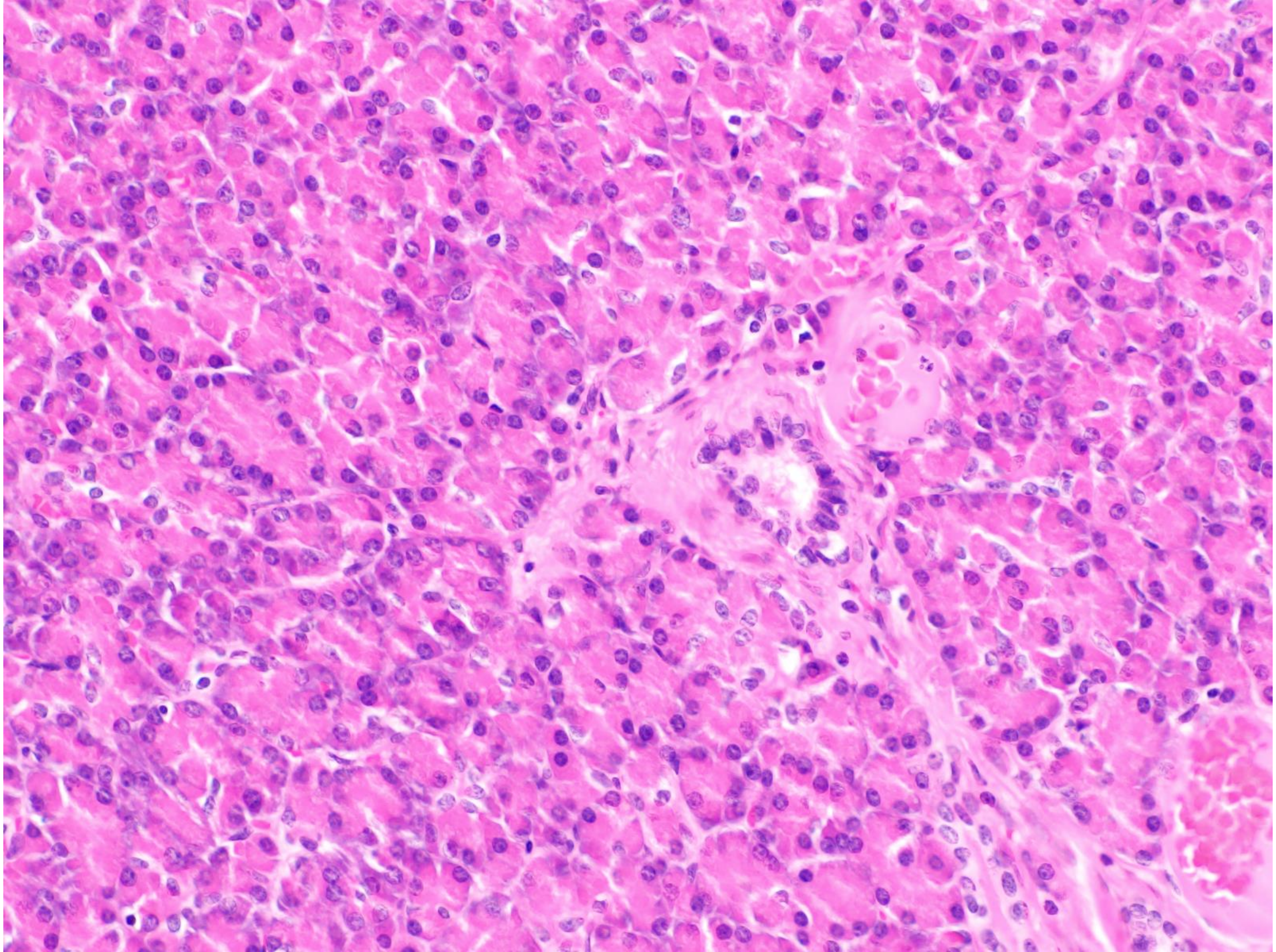

B

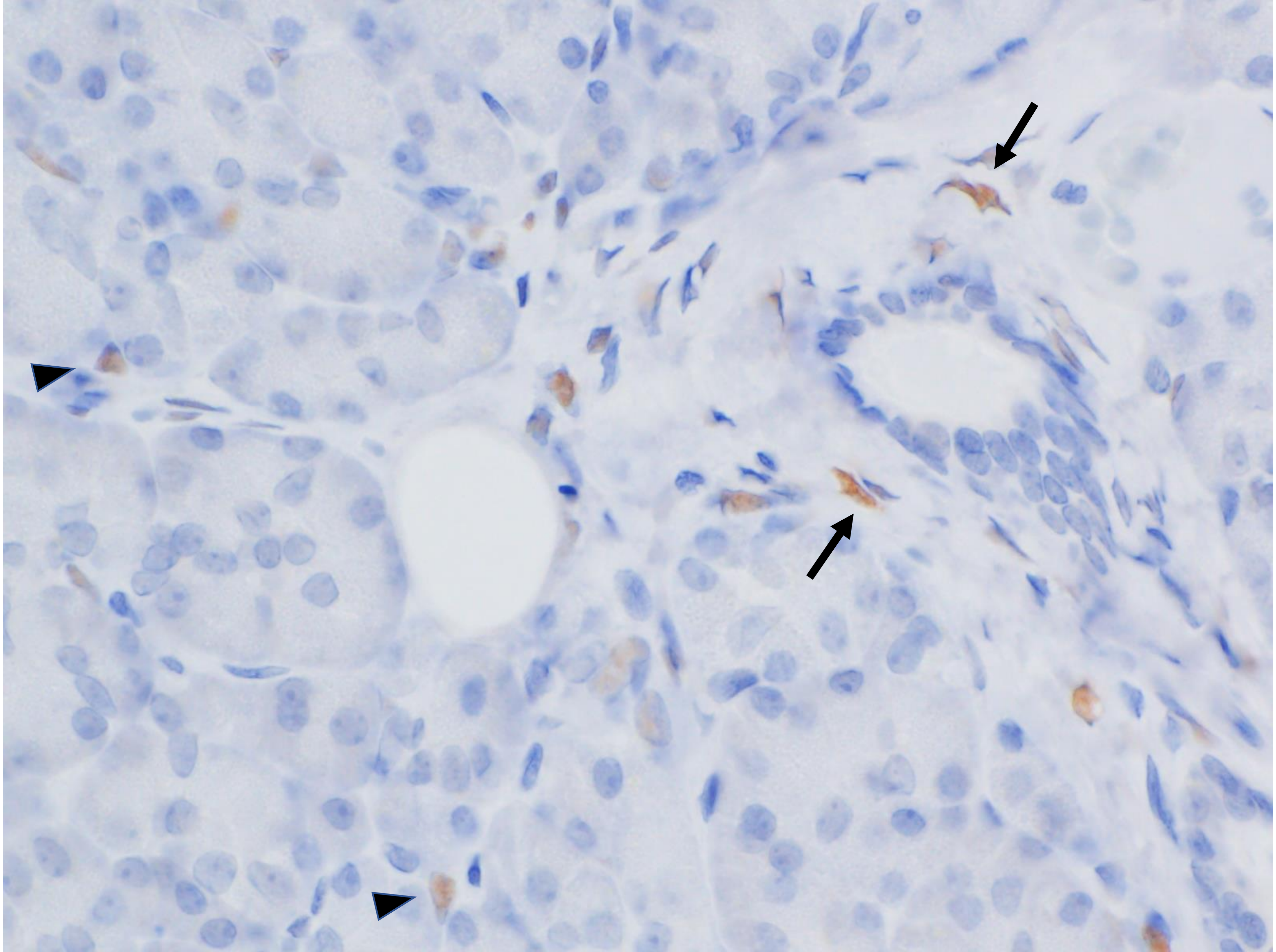

Supplement: Supplementary file 2 — Supplementary Information 2. [file 41598_2022_7416_MOESM2_ESM.pdf]
